# Realization and training of an inverter-based printed neuromorphic computing system

**DOI:** 10.1038/s41598-021-88396-0

**Published:** 2021-05-05

**Authors:** Dennis D. Weller, Michael Hefenbrock, Michael Beigl, Jasmin Aghassi-Hagmann, Mehdi B. Tahoori

**Affiliations:** 1 Chair of Dependable Nano Computing, Karlsruhe Institute of Technology, 76131 Karlsruhe, Germany; 2 Institute of Nanotechnology, Karlsruhe Institute of Technology, 76344 Eggenstein-Leopoldshafen, Germany; 3 Chair of Pervasive Computing Systems, Karlsruhe Institute of Technology, 76131 Karlsruhe, Germany; 4 Institute for Applied Research, Offenburg University of Applied Sciences, 77652 Offenburg, Germany

**Keywords:** Electrical and electronic engineering, Computer science

## Abstract

Emerging applications in soft robotics, wearables, smart consumer products or IoT-devices benefit from soft materials, flexible substrates in conjunction with electronic functionality. Due to high production costs and conformity restrictions, rigid silicon technologies do not meet application requirements in these new domains. However, whenever signal processing becomes too comprehensive, silicon technology must be used for the high-performance computing unit. At the same time, designing everything in flexible or printed electronics using conventional digital logic is not feasible yet due to the limitations of printed technologies in terms of performance, power and integration density. We propose to rather use the strengths of neuromorphic computing architectures consisting in their homogeneous topologies, few building blocks and analog signal processing to be mapped to an inkjet-printed hardware architecture. It has remained a challenge to demonstrate non-linear elements besides weighted aggregation. We demonstrate in this work printed hardware building blocks such as inverter-based comprehensive weight representation and resistive crossbars as well as printed transistor-based activation functions. In addition, we present a learning algorithm developed to train the proposed printed NCS architecture based on specific requirements and constraints of the technology.

## Introduction

With the recent advances of new functional materials, next-generation electronics, which can enable lightweight, cheap and flexible products are emerging. At the same time silicon computing systems still undergo continued improvements, which pushes the boundaries of performance, power consumption and transistor density of integrated circuits. The aforementioned requirements of emerging application domains can never be reached with fundamental silicon-based VLSI technology due to constraints such as bulky substrates and high production costs. Also innovative emerging technologies have opened a new market which enables penetrating our everyday-life by smart electronics in previously inaccessible application domains such as soft robotics^1^, soft sensors^2,3^, flexible wearable medical devices or Internet of things (IoT) infrastructures^4^. Especially for smart wearable devices and IoT, low-cost point-of-use fabrication techniques have to be explored to reduce the time-to-market of products which are part of the fast-moving consumer goods market^5^.

In order to enable this emerging field, new materials and processes are under development, which are expected to make the transition from research to application soon. In this regard, Printed Electronics (PE) is a promising candidate, as it offers ultra-low production costs due to its additive fabrication process^6^. Similar to color printing, PE fabrication processes can be categorized into different groups such as jet-printing^7,8^, screen printing^9^ or roll-to-roll^8^ processes. In contrast to the costly lithography processes for wafer-scale silicon-based ICs, these fabrication processes are highly simplified, as the functional materials can be deposited directly on a wide range of substrates, including flexible carrier materials^10–13^.

However, realization of printed smart electronics for sensor processing is not feasible yet in PE by using conventional digital computing. Due to the large feature sizes in PE, Boolean digital logic designs would lead to substantial area overhead, low performance and high power consumption, and consequently the aforementioned application requirements cannot be met^14^.

In this respect biological-inspired neuromorphic computing can be leveraged as a suitable computing paradigm for PE. A neuromorphic computing system (NCS) makes use of the physical characteristics of electronic devices, such as voltage-induced currents through electric resistors and is inspired by the topology of the brain. Instead of memory blocks and digital processing units, NCS is built from artificial synapses and neurons.

A neuromorphic computing system can directly process sensory data without converting it into digital signals, which otherwise would require expensive analog/digital converters (ADCs). Moreover, analog signal processing units lead to higher functional densities of printed circuits, and reduces both hardware footprint and power consumption of printed NCS (see Table 1). Finally, printed NCS can be trained to be intrinsically fault tolerant^15,16^, which increases the low chip yield in PE and thus ensures reliable operation.

Popular realizations of NCS are based on feed-forward/artificial neural networks (ANN)^17^. ANNs are akin to the popular neural networks developed for CPU- or GPU-platforms. Here, signals are represented by real-valued voltage signals and processing is performed by the McCulloch-Pitts neuron model^17^. Due to its simplicity, ANNs are commonly used in hardware implementations and training can be achieved by the well-known least-mean-square learning rule. Due to the back-propagation-based learning algorithm^18^, the training algorithm converges very fast to a solution and thus ANNs could demonstrate broad applicability^17^.

Very recently, NCS manufactured by additive technologies have been proposed. Basic synaptic functions were fabricated on flexible substrates^19^ using solution-based organic, electrochemically-gated transistors^20^ and screen-printing approaches with PEDOT:PSS as the active material^21^ as well as aerosol-jet-printing of carbon nanotube transistors (CNT)^22^. Moreover, a multiply-accumulate (MAC) engine on flexible substrates with a time domain encoded implementation was presented^23^. In^24^ an organic-based crossbar-architecture was introduced, which also realizes the MAC operation. Besides organic MAC operations, neuron activation functions were introduced. For instance, the authors of^25^ reported on a low-complexity design for activation function circuits based on organic p-type transistors for implementation of an ANN.

Although many of the aforementioned contributions realize parts of a printed NCS, they either do not provide all fundamental building blocks^24^, they are not based on printable materials^19,20,25^, or they can only be partially printed^21^. Also, the majority of existing solutions realize only MAC operations with positive neural network weights implemented by resistor- or memristor-based crossbar architectures^24–26^. However, this is a serious restriction as most neural network-based classifiers require also negative weights to achieve reasonable inference accuracy. Additionally, appropriate activation functions are required to provide full neuron processing. In fact, for many organic devices, the implementation of activation functions for ANNs - preferably consisting of several hidden layers—is not yet well-established^24^.

**Table 1:** Comparison between proposed ANN components (ADC: Analog–Digital-Converter, ReLU: Rectified Linear Unit) and conventional digital implementation.

Precision	Components	Delay (ms)	Area (${\hbox {mm}}^{2}$)	Power	#Transistors
4-bit	ADC	13.8	25.4	${328}\,{\upmu }\hbox {W}$	185
	Adder	13	7.9	${289}\,{\upmu }\hbox {W}$	59
	Multiplier	13.6	15	${550}\,{\upmu }\hbox {W}$	103
	ReLU	2.5	1.7	${80}\,{\upmu }\hbox {W}$	10
	Neuron	69	48	${1.25}\,{\hbox {mW}}$	357
8-bit	ADC	154	957	${37.18}\,{\hbox {mW}}$	5938
	Adder	29	22	${793}\,{\upmu }\hbox {W}$	144
	Multiplier	28	85	${3.1}\,{\hbox {mW}}$	583
	ReLU	2.55	3.7	${210}\,{\upmu }\hbox {W}$	22
	Neuron	522	1068	${41.25}\,{\hbox {mW}}$	6602
Analog	Neuron	27	0.49	${859}\,{\upmu }\hbox {W}$	4

The numbers for the neuron are for a 3-input neuron design. In the case of the digital implementation (4-bit and 8-bit), the computations (addition and multiplication) are performed sequentially.

**Figure 1:**
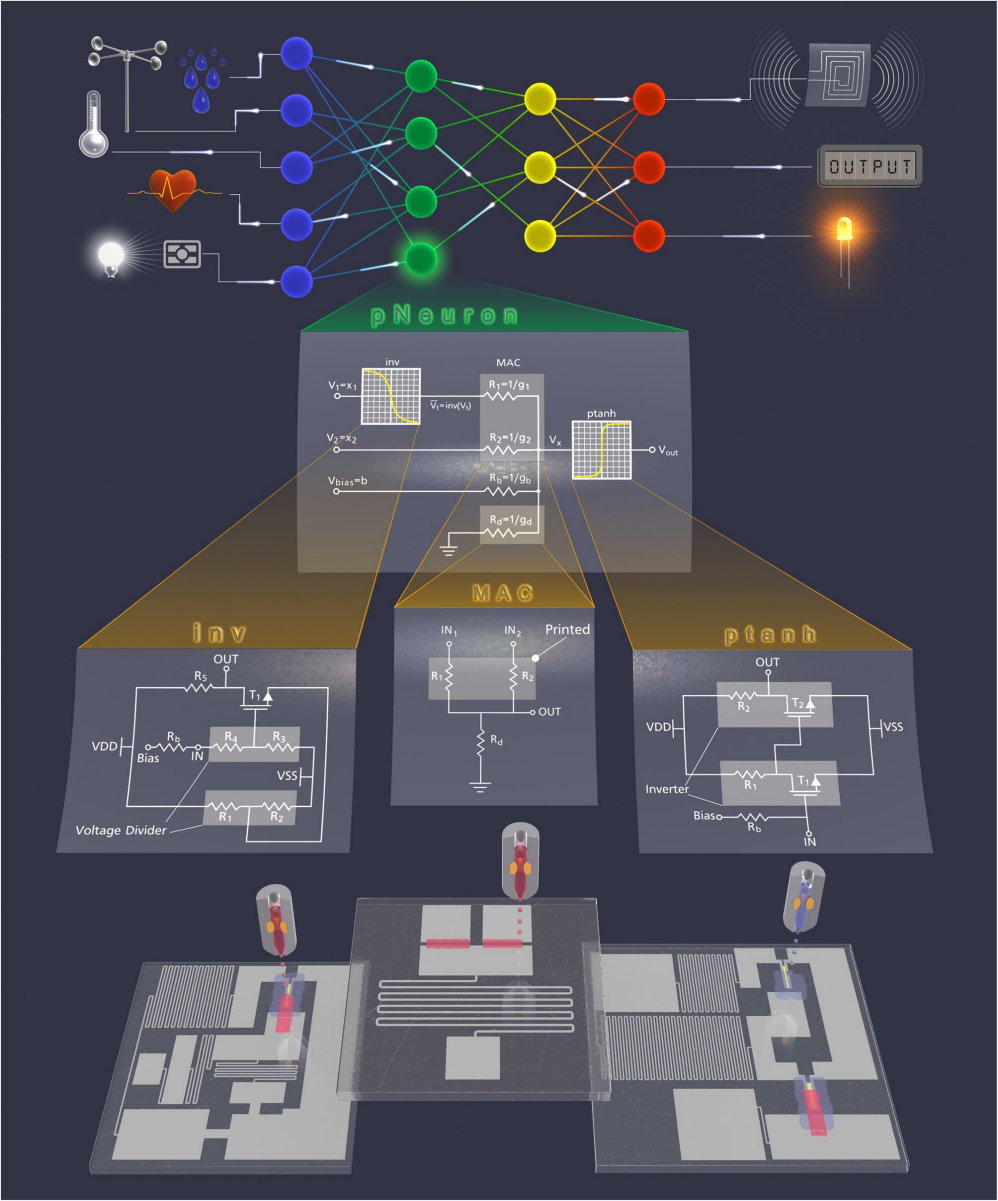
Top: Printed Neural Network with sensor inputs and actuators, Middle: circuit schematics of the inkjet-printed neuron (pNeuron) and the hardware prototypes of its three fundamental building blocks: negative weights circuit (inv), multiply accumulate operation (MAC) and non-linear tanh-like activation function (ptanh) Bottom: physical layout of the hardware prototypes.

In this work, we address the aforementioned shortcomings and demonstrate that all fundamental building blocks for printed ANNs can be fabricated within the same process and materials, such as MAC operation, non-linear activation functions and comprehensive weights representation. Among the different manufacturing processes, we deployed inkjet printing as the targeted fabrication technology in this work. Compared to roll-to-roll or screen-printing processes, inkjet-printing allows for highly customized designs, generated on-the-fly by a computer-aided design (CAD) software. This allows for on-demand designs and democratizes the fabrication for point-of-use hardware, enabling new use cases where silicon fabrication is not an option.

Prior work on inkjet-printed electronics were limited to standard building blocks, such as logic gates^27^, diode-based organic rectifiers^28^, amplifiers^29,30^, Digital-to-Analog converters^31^ and memories^32^. As a result, the proposed printed NCS presented in this work makes the transition from these conventional computing devices to inkjet-printed neuromorphic computing hardware.

The presented MAC operation in this work is realized by a crossbar architecture, where conductive materials vertically connect crossbar rows with columns to form artificial synapses. However, instead of using voltage-programmable memristors for the MAC operation^25^, printed resistors are used, which leads to several benefits: first, compared to memristors, resistors do not suffer from non-linear behavior, which would prevent back-propagation learning rules to converge^33^. Second, by printing resistors at the crossbar-interconnects, the circuit can be customized for diverse use cases for small and medium batch sizes.

Another contribution of this work is the adaption of ANN learning algorithms for NCS. As ANN learning routines are tailored to the functional behavior of MAC operations and activation functions, dissimilarities caused by different technologies prevent the application of existing learning solutions^34^ to converge. To this end, we provide a learning algorithm which takes these technology-specific ANN realizations into account, such as feasible ranges of printed resistors and incorporate the circuit-level characteristics of the printed ANN components to map the trained weights to the hardware level. Based on the target application and available datasets, the neural network weights are learned in a supervised training routine and printed to the ANN to achieve tailored signal processing. Moreover, due to the proposed variation-aware training methodology, the printed ANN is robust against process variation and substantially restores inference accuracy losses.

The proposed designs of the ANN components were validated by characterization of fabricated hardware prototypes, and in a second step a large-scale ANN was simulated and trained with the proposed learning routine and under consideration of the technology-dependent constraints. In summary, our approach proposes a NCS, which can be primarily deployed for near-sensor-processing and other application domains such as IoT and soft robotics.

## Results

### Proposed printed neuromorphic computing system

We will present in the following, designs and hardware prototypes of printed NCS components at the circuit level, which can be used as basic building blocks to realize arbitrary large ANN architectures in PE. These building blocks represent all three basic mathematical operations required to solve a wide range of ANN-based regression or classification tasks: MAC operation, positive and negative weights, and non-linear activation function.

The proposed NCS architecture is depicted in Fig. 1. At the top of Fig. 1, the high-level perspective of a printed NCS is illustrated. The printed NCS can be placed next to a sensor, from which it collects the input data. The sensory data is processed by the printed NCS using ANN inference. The ANN output signals can interface actuators for control commands, printed displays or wireless communication devices, to name a few examples.

While circuits for ANN operations can easily be derived for digital (Boolean) logic, analog designs require technology-dependent constraints. These constraints set bounds on maximum possible device performance, power consumption and area requirement, but also on device yield which limits the achievable complexity of printed analog hardware. As a result, analog building blocks such as operational amplifiers, which allow for realization of arithmetical operations are not feasible in PE, as they demand for many transistors.

Fortunately, as we demonstrate in this work, analog circuit designs exist which fulfill the aforementioned requirements and at the same time provide all the functionalities to map neural network concepts with all its basic building blocks to PE.

For general neural networks running on CPUs or GPUs, the negative weights operation is not termed as an individual feature for NNs as it is a part of the MAC operation, which is implemented in software and performed by the floating-point unit of the processor. As we neither have a floating-point unit in PE, nor a microprocessor which carries out software programs, an alternative must be found for PE circuits.

All the designs of the three building blocks presented in this work, have the capability to process analog data, encoded by voltage levels. These physical quantities with their continuous value representation are a substitution of digital multi-bit logic, which would lead to infeasible design overhead in PE. Moreover, the presented building blocks have very low device count, not more than two transistors per device, which is even exceptionally in the analog electronics domain.

As illustrated in Fig. 1, by interconnection of the three building blocks (“inv”, “MAC”, “ptanh”), one neuron (“pNeuron”) can be implemented with arbitrary number of input signals. By interconnection of different neurons, a larger scale artificial neural network can be realized (top of Fig. 1). These three fundamental building blocks are described separately in the following.

#### Multiply accumulate (MAC) operation

The MAC operation is a standard operation for performing ANN inference. At each ANN node, the MAC operation—as part of a single neuron—adds up the inputs, scaled and weighted by the ANN weights $w_i$. The output of the MAC operation can be computed by:

1 $$\begin{aligned} a = \mathrm {\mathbf {x}}^T\mathrm {\mathbf {w}} = \sum _i w_i x_i + w_b x_b = \sum _i w_i x_i + b \end{aligned}$$

where $x_b$ is a constant input and thus $w_b x_b$ is denoted as the constant neuron bias *b*.

**Figure 2:**
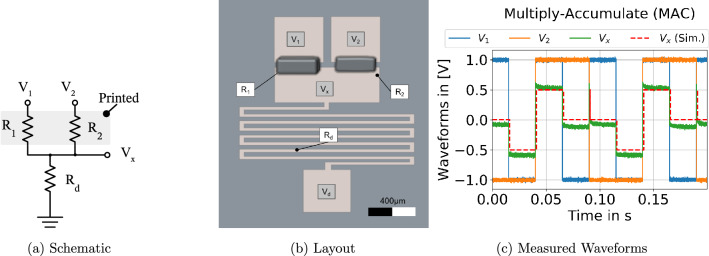
(**a**) The schematic of the 2-input crossbar ($V_1, V_2$) implementing the MAC operation. (**b**) Depicts the layout of the fabricated MAC circuit. (**c**) Illustrates the waveforms from the simulation and circuit measurements.

For ANNs implemented in software and running on CPUs, this operation is usually performed sequentially in the floating-point unit, or in parallel on Digital Signal Processors (DSP). In contrast, the implementation of MACs for printed neuromorphic hardware is much different. First of all, the inputs of a neuron $x_i$ are encoded as voltage signals $V_i$ (or $\bar{V}_i$ if the input is inverted), which are applied to a crossbar architecture with printed resistors $R_i$ at the crossbar interconnects (see Fig. 2a). According to Ohm’s law, the voltages across the resistors ($V_i - V_x$) are converted into currents, and these currents are summed up following Kirchhoff’s rule. The output voltage $V_x$ of the crossbar can be computed similar to a Y-circuit (i.e., a circuit where one port of each resistor is connected together). As only resistors are contained, the crossbar resembles a linear circuit and the output voltage $V_x$ can be computed analytically (a more detailed mathematical derivative of the MAC formulas is provided in the supplementary document).

The relationship between the neuron input voltages $x_i = V_x$ and the neuron weights $w_i$, as well as the constant bias voltage $b = V_{bias} \cdot \ w_b$, is as follows:

2 $$\begin{aligned} {\begin{matrix} V_x&= \sum _i V_i \ w_i + V_{bias} \ w_b \end{matrix}} \end{aligned}$$

For simplicity, we denote in the following the resistors $R_i$ with the conductances $g_i = \frac{1}{R_i}$, or $g_b = \frac{1}{R_b}$ and $g_d = \frac{1}{R_d}$ respectively.

Thus, the synaptic weights can be abbreviated by:

3 $$\begin{aligned} w_i = \dfrac{g_i}{\left( \sum _j g_j\right) + g_b + g_d} \end{aligned}$$

and for the bias weight:

4 $$\begin{aligned} w_b = \dfrac{g_b}{\left( \sum _j g_j\right) + g_b + g_d} \end{aligned}$$

and the decoupling weight:

5 $$\begin{aligned} w_d = \dfrac{g_d}{\left( \sum _j g_j\right) + g_b + g_d} \end{aligned}$$

As can be obtained from Eq. (2), the crossbar output $V_x$ behaves like a MAC operation:

6 $$\begin{aligned} a = V_x = \sum _i w_i \ V_i + w_b \ V_{bias} = \sum _i w_i x_i + b \end{aligned}$$

Moreover, from Eqs. (3) and (4) it is obvious that $w_i$ and $w_b$ are lower- and upper-bounded: $w_i,w_b \in [0,1]$. A reason for the lower bound is the fact that resistors are physically only positive. On the other side, the upper bound can be explained that in a passive resistor network, applied voltages cannot be increased, only reduced due to power dissipation. Another constraint can be obtained by summing up all $w_i$, $w_b$ and $w_d$, as a result the second constraint is:

7 $$\begin{aligned} \sum _i w_i + w_b + w_d = 1 \end{aligned}$$

It is important to note that the above Eq. (7) with coupled weights can be decoupled by proper adjustment of the conductor/resistor $g_d$/$R_d$ (also called $R_{base}$, see^25^). The decoupling resistor $R_d$ is added to the resistive crossbar similar to $R_i$ and $R_b$, however a constant voltage of 0 V is applied to it (i.e. $V_d = 0$ V), see Fig. 1. As a result, the decoupling resistor can be used as a placeholder to adjust the weight formula (Eqs. (3) and (4)) without biasing the MAC output $V_x$ (Eq. (2)).

Although decoupling of weights through $g_d$ simplifies ANN training, as (one constraint less), it leads to smaller absolute values of all other $w_i$ (second constraint), which implies high signal losses at each ANN layer and consequently the output signals of the crossbar—the result of the MAC operation—becomes susceptible to signal noise. However, this voltage degradation can be compensated by the activation function proposed in this work, which despite their non-linear property also behave as voltage buffer elements.

In Fig. 2a the schematic of the programmable resistor-based crossbar is illustrated with two inputs $V_1$ and $V_2$, printed crossbar-resistances $R_1$, $R_2$, and decoupling resistor $R_d$. It is important to mention that for the realization of a negative weight a single transistor is required, otherwise the MAC operation can be implemented by a passive circuit only consisting of resistors.

#### Negative weights operation (inv)

The MAC operation in a single printed neuron can perform vector operations between a variable input signal vector ${\varvec{x}}$ and a constant weight vector ${\varvec{w}}$. However, due to the implementation by a resistor crossbar, negative multiplications between inputs $x_i$ and weights $w_i$ (e.g., $w_i < 0$) are not possible, as the resistances are physically only positive and the weights/resistor dependency is proportional (see Eqs. (3) and (4)). The restriction to only positive weights yields an ANN classifier whose output function is intrinsically monotonic in relation to its inputs. This would limit the applicability to potential classification or regression problems.

**Figure 3:**
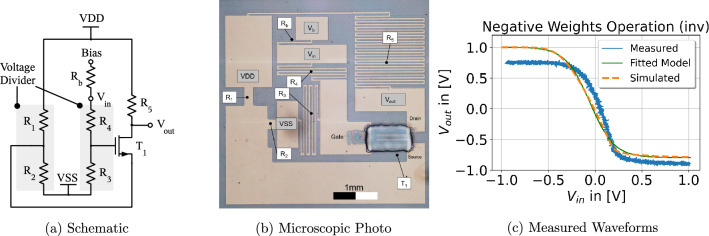
(**a**) The schematic of the proposed negative weight circuit. Two voltage dividers are deployed to shift the zero-crossing of the output voltage towards ${0}{\hbox {V}}$. (**b**) Depicts the microscopic photo of the hardware prototype. (**c**) Contains the simulated and measured transfer function of the circuit.

In order to achieve negative weights, we propose an inverter-based transfer function (Fig. 3a), which turns positive neuron input voltages into negative voltages and vice versa, similar to the operation $x_i \cdot (-1)$. The benefit compared to other existing techniques^34^ is that the negative weights circuit is only used when necessary and then placed before crossbar resistors whose weights should be negated (Fig. 1). Consequently, the resulting printed neuron design offers less area, significantly less power consumption and reduced transistor count. Moreover, less material has to be printed which also reduces printing costs and time.

#### Tanh-like activation function (ptanh)

**Figure 4:**
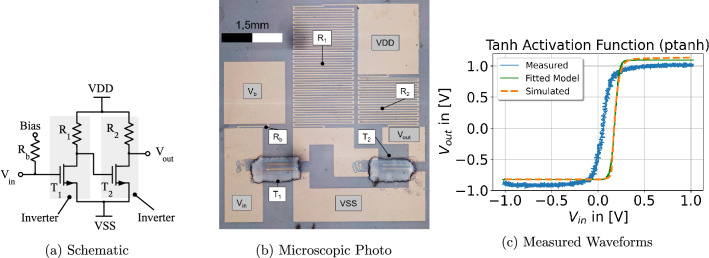
(**a**) Schematic of inverter-based activation function for realizing tanh function (**b**) depicts a microscopic photo of the fabricated hardware prototype of (ptanh) (**c**) contains the simulated and measured waveforms.

A non-linear activation is the third component required for ANN computations^35^. This operation is applied directly to the output of the MAC operation (see Fig. 1). Problems with previous presented printable activation functions^25,26^ are the induced signal losses at the layer output, and moreover the lack of amplification possibilities between layers. A more suitable and feasible choice of an activation function circuit is the inverter-based approach presented in Fig. 4a. By cascading of two inverters, it acts as a non-inverting buffer element, which in addition to its non-linear behavior, restores voltage levels between the ANN layers. The behavior of this circuit resembles a hyperbolic tangent (tanh), thus we abbreviate it in the following as ptanh (printed tanh). The transfer function of the circuit is depicted in Figs. 1 and 4c.

#### Printed neural network

By interconnection of the three ANN building blocks, a printed functional neuron (pNeuron) can be constructed, with arbitrary number of neural inputs (i.e., synapses). It is remarkable that the pNeuron requires only two transistors for the activation function, and one transistor for each negative weight operation. This leads to very low hardware footprint and small delay and power consumption in comparison with a conventional digital implementation.

This fact can also be derived from Table 1, where we compared an analog 3-input neuron with a 4-bit and 8-bit digital implementation. The digital neuron deploys a fixed-point multiplication unit with a rectified linear unit (ReLU) as the activation function. In order to keep the number of transistors reasonable, the multiplications and additions of the digital 3-input neuron are performed sequentially.

For the digital components, high-level synthesis tools were deployed to extract circuit characteristics. Due to the large number of transistors caused by the implementations of expensive analog-digital-converters (ADC) and multiplier units, the digital designs are infeasible to be fabricated by the inkjet-printing technology. When increasing the precision of the digital circuits from 4-bit to 8-bit, a trend of exponentially increasing transistor count, area, delay and power consumption can be observed. Moreover, as can be obtained from Table 1, the analog implementation requires much less area and is also superior in terms of delay and power consumption in comparison to the 4-bit and 8-bit digital neuron implementation. Obviously, this analysis encourages the utilization of analog designs in PE for NCS realization.

Based on the pNeuron design, larger NNs can be constructed by replicating the proposed neurons intra- and inter-layer-wise (Fig. 1 top). The resulting ANN can be deep with many hidden layers due to the amplification of the ANN outputs at the non-linear activation function. Furthermore, the printed NCS can be tailored to a target application, by first choosing the ANN topology, number of nodes and layers, and second by programming the MAC operation by resistor printing to the crossbar interconnects after ANN training for point-of-use customization. As the negative weights circuit is only inserted at the required locations, this leads to a sparse ANN implementation with fewer components compared to existing designs^34^.

### Hardware prototypes characterization

As a proof of concept, the three building blocks for printed NCS were fabricated using an Fujifilm Dimatix DMP 2850 material inkjet printer. The corresponding circuit diagrams, layouts/microscope photos and measured transfer functions of the three hardware prototypes of the fundamental ANN building blocks are depicted in Fig. 2 (MAC), Figs. 3 (inv) and 4 (ptanh).

#### Multiply-accumulate (MAC) prototype

In Fig. 2a the circuit for the MAC operation is depicted. The weights $w_i$ of the MAC operation depend on the resistance vales of $R_1$ and $R_2$, which are inkjet-printed and customized according to the ANN training step. Figure 2b shows the layout of the hardware prototype of the printed crossbar. The passive conductive structures, including the meander resistor ($R_d$) were obtained from laser-ablated ITO-sputtered glass substrates. The resistors $R_1$ and $R_2$ were printed using PEDOT:PSS conductive ink. This allows for customization of the neuron according to the pre-trained weights vectors.

The transient measured input and output waveforms are depicted in Fig. 2c. Both $V_1$ and $V_2$ are pulsed between ${-1}$ and ${1}\,{\hbox {V}}$, with a pulse width of ${10}\,{\hbox {ms}}$ and ${5}\,{\hbox {ms}}$, respectively. The output pulse $V_x$ is obtained in dependence of the input pulses. The circuit response behaves as expected, showing only close to ${0}{\hbox {V}}$ signals when the inputs are complementary to each other. The output $V_x$ is pulled up or down to ${0.5}\, {\hbox {V}}$ or ${-\,0.5}\, {\hbox {V}}$ when both signals are at ${1}\,{\hbox {V}}$ or ${-1}\,{\hbox {V}}$, respectively. The resulting weights were: $w_1 = w_2 \approx 0.25$ and $w_d \approx 0.5$. So both coefficients of the MAC operation are set to 0.25, and the correct summation and multiplication result $V_x = w_1 \cdot V_1 + w_2 \cdot V_2 = 0.25 \cdot V_1 + 0.25 \cdot V_2$ is obtained from the measured waveform. Figure 2c also shows the simulated waveform extracted from the circuit simulator. The measured signal is approaching the simulation result and confirms the correct implementation of the MAC circuit.

The choice of design parameters and the measurement results are depicted in Table 2.

**Table 2:** Design parameters and result metrics of hardware prototypes.

Components		MAC	inv	ptanh
Design parameters		$R_1$ = ${100}\,{\hbox {k}}{\Omega }$	$R_1/R_2$ = ${160}\,{\Omega }$/${80}\,{\Omega }$	$R_1/R_2$ = ${180}\,{\hbox {k}}{\Omega }$/${80}\,{\hbox {k}}{\Omega }$
		$R_2$ = ${100}\,{\hbox {k}}{\Omega }$	$R_3/R_4$ = ${25}\,{\hbox {k}}{\Omega }$/${15}\,{\hbox {k}}{\Omega }$	$T_1$ = ${100}\,{\upmu \hbox {m}}$/${80}\,{\upmu \hbox {m}}$
		$R_d$ = ${50}\,{\hbox {k}}{\Omega }$	$R_5$ = ${80}\,{\hbox {k}}{\Omega }$; $T_1$ = ${500}\,{\upmu \hbox {m}}$/${40}\,{\upmu \hbox {m}}$	$T_2$ = ${500}\,{\upmu \hbox {m}}$/${40}\,{\upmu \hbox {m}}$
			VDD/VSS = ${1}\,{\hbox {V}}$/${-\,2.2}\,{\hbox {V}}$	VDD/VSS = ${1}\,{\hbox {V}}$/${-\,1}\,{\hbox {V}}$
Results	Delay	${1}\,{\hbox {ms}}$	${4}\,{\hbox {ms}}$	${4.5}\,{\hbox {ms}}$
	Power	${31.3}\,{\upmu }\hbox {W}$	${30}\,{\hbox {mW}}$	${44.8}\,{\upmu }\hbox {W}$
	Area	${2.6}\,{\hbox {mm}}^{2}$	${20.3}\,{\hbox {mm}}^{2}$	${25.6}\,{\hbox {mm}}^{2}$

Resistance values $R_i$,$R_d$ are indicated, also the supply voltages VDD/VSS and the printed transistor channel geometries $T_i$=width/length=W/L.

#### Negative weights prototype (inv)

The proposed circuit schematic of the negative weight operation is shown in Fig. 3a. Due to the absence of PMOS transistors in this technology, the pullup network of the inverter consists of a resistor ($R_5$) and an NMOS transistor in the pull-down network ($T_1$). Moreover, two voltage dividers ($R_1$,$R_2$ and $R_3$,$R_4$) are inserted to improve the rail-to-rail behaviour. The additional bias pin was inserted for fine-tuning and shifting of the zero crossing point but was however not required in the final experiment.

The resistor and transistor sizing were performed using SPICE simulations based on a prior developed printed process design kit (PPDK)^36^. The design parameter values are provided in Table 2. VSS was adjusted from initial ${-2.0}{\hbox {V}}$ to ${-2.2}{\hbox {V}}$ to shift the zero crossing point of the circuit transfer function towards ${0}{\hbox {V}}$.

The output transfer function’s range of values is approximately between ${-1}{\hbox {V}}$ and ${1}{\hbox {V}}$, thus signal degradation across this circuit element is prevented as the full input voltage swing is also provided at the output.

The DC measurements of the negative weights circuit are shown in Fig. 3c, where the input signal $V_{in}$ is swept from ${-1}{\hbox {V}}$ to ${1}{\hbox {V}}$. It can be seen that the output signal $V_{out}$ follows the input signal $V_{in}$ inversely proportional. As we show later, this behaviour is adequate to approximate negative ANN weights which allow for successful classification of benchmark datasets. The measured and simulated DC transfer functions of the negative weights circuit are depicted in Fig. 3c. The simulated transfer function used for the high-level benchmark dataset evaluation is parameterized by the following model function (this fit is also depicted in Fig. 3c):

8 $$\begin{aligned} {\text {inv}}(x) = -(\eta _1 + \eta _2 \cdot \tanh ((x - \eta _3) \cdot \eta _4 )) \end{aligned}$$

with

$$\eta _1 = -0.104,\ \eta _2 = 0.899,\ \eta _3 = -0.056,\ \eta _4 = 3.858$$

As the tanh function is a point symmetric function, multiple parameters ${\varvec{\eta }}$ exist which implement the same functionality. For this reason, we bounded the parameter $\eta _4$ to be positive to obtain a unique parametrization. The choice of design parameters and the measurement results are depicted in Table 2. The reason why the power consumption of this circuit is so high is due to the first voltage divider $R_1$ and $R_2$ . As the resistances of both resistors are in the range of only hundreds of ohms and as secondly a constant voltage source is applied to them (VDD-VSS), a high current is induced which leads to high power dissipation. One way to achieve substantial power consumption reduction is by scaling all resistances $R_1$-$R_4$ up by a constant factor $\gg 1$.

#### Tanh-like activation function prototype (ptanh)

The schematic of the tanh-like function is depicted in Fig. 4a. Resistor sizing was achieved by design extraction based on SPICE simulations and the PPDK. A microscope photo is shown in Fig. 4b. Two transistors were printed, one for each inverter, and the conductive tracks were again obtained from laser-structured ITO-sputtered glass substrates. The design parameters chosen for this component are illustrated in Table 2. The additional bias pin for zero crossing point tuning was not required in this experiment.

The measured and simulated DC transfer functions of the ptanh circuit are depicted in Fig. 4c. As can be seen, excellent rail-to-rail behavior is observed. The output signal voltage levels are ideally pulled up/down to ${1}\,{\hbox {V}}$/${-1}\,{\hbox {V}}$, thus this component can preferably deployed as a voltage buffer for voltage signal replenishment at the output of each ANN layer. An important remark here is that the sensing resolution of the output signal was about ${100}\,{\hbox {mV}}$. In order to be able to distinguish the low- and high output signals of this activation function, we introduce a safety margin through the learning function of the ANN (more details in the Supplementary Document).

The simulated transfer function used for the high-level benchmark dataset evaluation is parameterized by the following model function:

9 $$\begin{aligned} {\text {ptanh}}(x) = \eta _1 + \eta _2 \cdot \tanh ((x - \eta _3) \cdot \eta _4 ) \end{aligned}$$

with

$$\begin{aligned} \eta _1 = 0.134,\ \eta _2 = 0.962,\ \eta _3 = 0.183,\ \eta _4 = 24.10. \end{aligned}$$

Similar to the fit of the ${\text {inv}}(x)$ function, the parameter $\eta _4$ is bounded to the positive range to obtain a unique parametrization. The function fit is also indicated in Fig. 4c. The measurement results about delay, power and area are presented in Table 2.

### Training printed neural networks

To train the proposed printed neural networks, several steps of the classical (hardware-agnostic) neural network training procedure need to be adapted to consider the technological constraints regarding the ranges of feasible resistances. Additionally, the circuit level constraints, such as the coupling of the weight values need to be respected. Finally, separability of the voltage signals at the ANN output layer has to be considered to obtain the correct classification outcome. This requires a minimum sensing resolution of the output signals, which has to be considered during training.

These constraints are addressed by introducing special parameters we call surrogate conductances $\theta _i$. The surrogate conductances encode the value of a respective conductance through their absolute value, i.e., $g_i = \; \mid \theta _i \mid$ while the sign of $\theta _i$ encodes if the input to the respective resistor should be inverted (negative weight). Through this, the weighted sum (or MAC operation) is modelled as

$$\begin{aligned} \sum _i w_i \left( x_i\cdot \mathbb{1}_{\left\{ \theta _i \; \ge \;0\right\} } + {\text {inv}}(x_i)\cdot \mathbb{1}_{\left\{ \theta _i \; < \; 0\right\} } \right) , \end{aligned}$$

where ${\text {inv}}(x)$ denotes the inverted input *x* (see Fig. 3c for the graph of ${\text {inv}}(x)$) and $\mathbb{1}_{\left\{ \cdot \right\} }$ denotes an indicator function returning 1 if the respective condition is true, else 0.

Furthermore, the signal separation is encouraged through a custom loss function that is further enhanced through a penalty term. The penalty term is thereby introduced to penalize infeasible conductance values in training which is performed using the backpropagation ^18^ algorithm. To stabilize the training, the initial values for the training variables are chosen in consideration of the characteristics of the printed tanh (ptanh) activation function. After the training is complete, infeasibly small conductance values are set to zero. This relates to not printing the respective connection.

Many sources of variation, such as printed conductance and transistor variations as well as voltage and temperature fluctuations or variations in the activation and inverter function circuit may impair the performance and accuracy of the printed neural networks. To combat these effects, we propose a variation-aware training solution. Here, the training objective is to minimize the expected loss under simulated variations by using Monte Carlo gradient estimates^37^ for backpropagation^18^. For the details of the specific steps, see Sects. 3 and 4 of the supplementary material.

### The entire design flow and training

**Figure 5:**
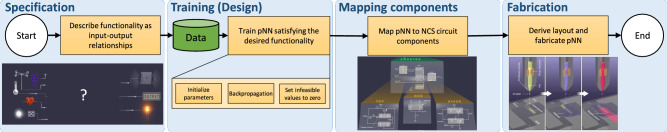
The proposed procedure allows for an on-demand design and fabrication given a specification of a desired functionality. While the on-demand design is realized through training a pNN according to the specification, the derived design can be readily fabricated through the on-demand fabrication capabilities of inkjet-printed electronics.

The complete flow of design, training, mapping and fabrication can be seen in flowchart of Fig. 5. From an initial idea, the desired functionality is described in the form of input-output relationships. These relationships are then converted into training data for the printed neural network (pNN). Through the training procedure, a pNN is developed expressing the desired functionality. Then the automatic circuit design is performed where the trained pNN is mapped into printed hardware components and their interconnections. In the last step, the circuit realizing the desired functionality is fabricated.

### Benchmark results for trained printed neural networks

To validate that our pNN is trainable, robust and sufficiently expressive under the given restrictions, we trained and evaluated several pNNs using our proposed variation-aware training procedure. The results of the learned pNNs are compared to a random guess baseline (most frequent training data class) and a standard (hardware-agnostic) neural network implementation of a comparable architecture. This network is referred to as reference NN in the following. For the reference NN, a standard *tanh* activation function is used instead of the ptanh activation function.

In order to assess the efficiency of our variation-aware training method, we performed PVT (process, voltage, temperature) variation analysis on both a nominal-trained pNN and the variation-aware trained NN. The results are discussed in the following. For a detailed discussion of the training process and preparation of the data see the Supplementary Material.

#### PVT-analysis on nominal-trained pNN

**Figure 6:**
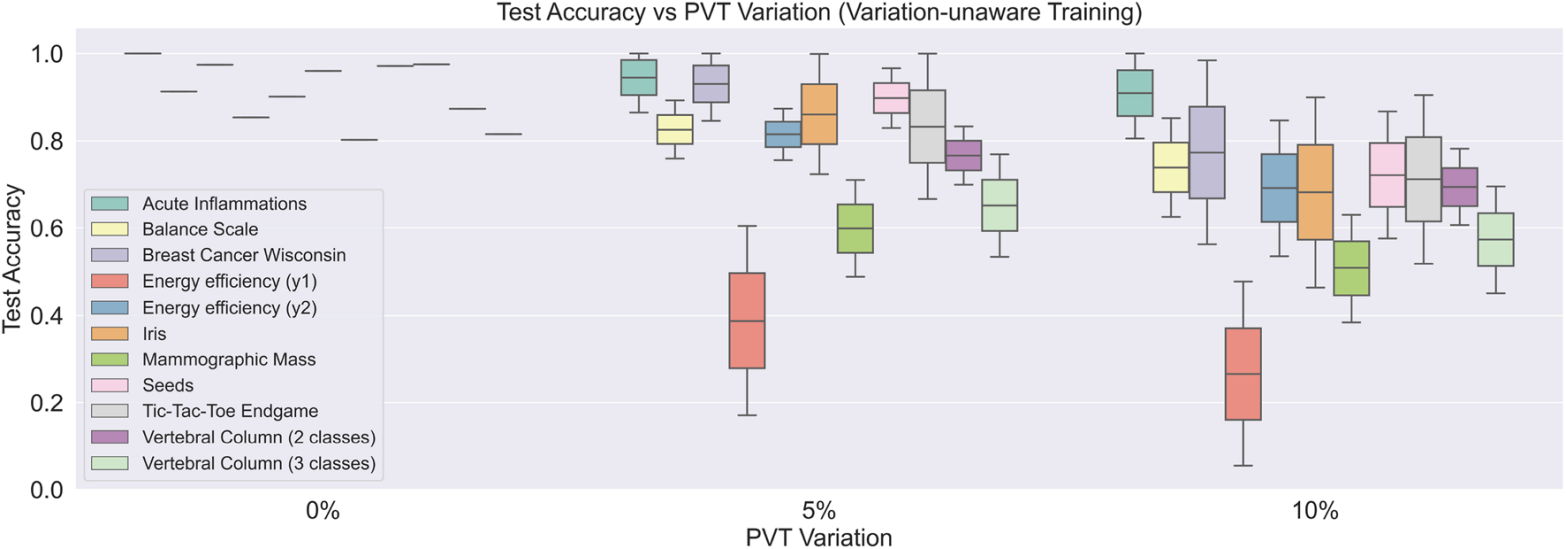
PVT analysis—inference accuracy of pNN using nominal (variation-unaware) training procedure. Variation parameter represents the coefficient of variation of the process, voltage and temperature variation models. The middle lines in the boxes refer to the mean test accuracy, while the whiskers depict the standard deviation.

For the PVT analysis, we used variation models of the pNN circuit components as discussed in Sect. 4.5 in the Supplementary Material. Monte Carlo simulations were performed during NN inference based on the pNN variation model and coefficient of variation. The impact of PVT variation on the nominal-trained pNN are depicted in the barchart of Fig. 6. As can be obtained from Fig. 6, the impact of PVT variations on the inference accuracy is dataset-dependent. While most of the Monte Carlo samples for ’Acute Inflammations’ achieve a test accuracy above 90%, samples for ’Energy efficiency (y1)’ reach from 60% down to less than 20%. For 10% (coefficient of variation) PVT variation, the test accuracies are wider distributed, and mean accuracy is below 75% for the majority of the datasets. The PVT variation results for the nominal-trained pNNs are also presented in Table 3.

#### PVT-analysis on variation-aware-trained pNN

**Figure 7:**
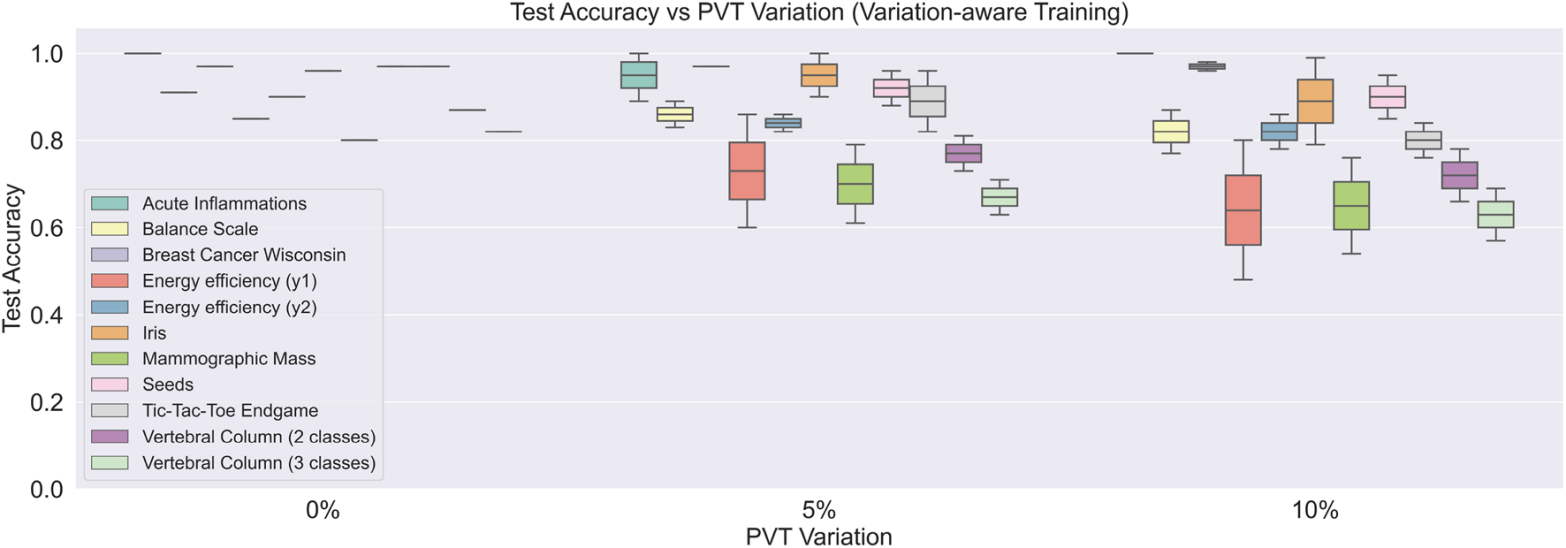
PVT analysis—inference accuracy of pNN using variation-aware training procedure. Variation parameter represents the coefficient of variation of the process, voltage and temperature variation models. The middle lines in the boxes refer to the mean test accuracy, while the whiskers depict the standard deviation.

The PVT-variation-based Monte Carlo simulation for the variation-aware-trained pNN are illustrated in Fig. 7. From the figure the fact can be derived, that the variation-aware training method substantially improves the test accuracy. For both, 5% and 10% PVT variation, the test accuracy distributions are much narrower. Also for 5% PVT variation, the mean test accuracies are above 80% for most of the datasets. The impact of variation-aware training is still existent for 10% PVT variation.

The PVT variation results are also summarized in Table 3, which additionally contains the standard (hardware-agnostic) NN, the variation-free pNN (0% variation) and the random guess baseline. Instead of the conventionally used standard inference accuracy, we deploy measuring-aware accuracy (MaA) as the evaluation metric, as it takes also the differentiability of measured output voltages of the pNN into account, which is important in a practical case involving a measurement tool or actuators at the pNN output layers which require a particular sensing margin *T*. The proposed evaluation metric is described in more detail in Sect. 5 in the Supplementary document.

Regarding the simulation results in Table 3, our main observation is that all pNNs surpass the baseline result. Secondly, we can see that for the variation-aware pNN almost all the trained pNNs reach comparable performance to the reference network. Thirdly, the variation-aware-trained pNN has acceptable test accuracy for the 5% PVT variation corner for the majority of datasets. We therefore conclude that the proposed pNN models and designs are robust against variations, including process variations leading to imperfections of the ’inv’ and ’ptanh’ building blocks.

**Table 3:** The evaluation table of the experiments.

Dataset	Architecture	pNN (variation-aware)			pNN (nominal)		Reference NN	Random guess
		0%	5%	10%	5%	10%		
Acute inflammations	6-4-3-2	1	0.95 ± 0.06	1.0 ± 0.00	0.945 ± 0.08	0.91 ± 0.1	1	0.48
Balance Scale	4-4-3-3	0.91	0.86 ± 0.03	0.82 ± 0.05	0.83 ± 0.07	0.74 ± 0.11	0.89	0.44
Breast cancer Wisconsin	9-4-3-2	0.97	0.97 ± 0.0	0.97 ± 0.01	0.93 ± 0.08	0.77 ± 0.21	0.97	0.67
Energy efficiency (y1)	8-4-3-3	0.85	0.73 ± 0.13	0.64 ± 0.16	0.387 ± 0.22	0.27 ± 0.21	0.86	0.43
Energy efficiency (y2)	8-4-3-3	0.90	0.84 ± 0.02	0.82 ± 0.04	0.82 ± 0.06	0.7 ± 0.16	0.91	0.46
Iris	4-4-3-3	0.96	0.95 ± 0.05	0.89 ± 0.10	0.86 ± 0.14	0.68 ± 0.22	0.98	0.28
Mammographic mass	5-4-3-2	0.80	0.70 ± 0.09	0.65 ± 0.11	0.6 ± 0.11	0.51 ± 0.12	0.81	0.55
Seeds	7-4-3-3	0.97	0.92 ± 0.04	0.90 ± 0.05	0.9 ± 0.07	0.72 ± 0.15	0.96	0.27
Tic-Tac-Toe Endgame	9-4-3-2	0.97	0.89 ± 0.07	0.80 ± 0.04	0.83 ± 0.17	0.71 ± 0.2	0.97	0.64
Vertebral Column (2 classes)	6-4-3-2	0.87	0.77 ± 0.04	0.72 ± 0.06	0.77 ± 0.07	0.7 ± 0.09	0.88	0.69
Vertebral Column (3 classes)	6-4-3-3	0.82	0.67 ± 0.04	0.63 ± 0.06	0.65 ± 0.12	0.57 ± 0.12	0.79	0.51

Architecture with neurons/layer. The experiments were carried out as described in Sect. 5 of the supplementary material. Each number represents test measuring-aware accuracy (see supplementary material). The results under variation are based on 100 samples and the mean and the standard deviation of the Measuring-aware accuracy are reported. The reported random guess baseline indicates the accuracy for always predicting the most frequent class in the training set.

### Discussion/conclusion

In this work, we propose the architecture and implementation of a NCS realized by inkjet-printed metal oxide electronics, which allows for flexible form factors and enables customized solutions for future applications. The presented neuromorphic hardware solves classification problems, which is in particularly interesting for near-sensor processing applications and smart products of low-cost consumer markets.

The presented NCS is built from functional materials, which can all be deposited by a compact and low-cost inkjet printer, leading to very low fabrication costs and enables new solutions of customized electronics which cannot be realized by silicon-based processes. Besides the technology, we propose circuit designs for this technology, which provide all functional operations to implement non-spiking artificial neural networks. Several constraints from the technology and printed neuromorphic architecture are considered, such as limited set of digital and analog components in PE, and signal degradation across ANN layers. As a proof of concept, the proposed circuit designs for printed NCS were fabricated using an Dimatix inkjet printer.

A significant advantage of our approach is that arbitrary large printed (deep) neural networks can be built due to the signal restoration property of the printed neuron concept. The ANN is also scalable with respect to the ANN layer size due to the crossbar-based multiply accumulate operation. The proposed ANN architecture is validated simulation-based on benchmark datasets of popular classification problems. PVT analysis was performed to prove that the variation-aware trained pNN is also robust against variations including imperfections of the fabricated ANN building blocks. In this regard, we propose a variation-aware training routine which can be deployed before pNN fabrication and which in particular takes the process-induced pNN component variations into account, enabling high-accuracy neural network classifiers in PE.

The area requirement of the ANN in this work was about ${400}{\hbox {mm}}^{2}$ with a delay of ${30}{\hbox {ms}}$ during ANN inference. However, extreme improvements in area, delay and power are expected in future designs which are beyond the capabilities of the presented hardware prototypes in this work. Further developments of the presented printing technology are replacement of the conductive tracks by inkjet-printed conductors or even by screen-printed materials to maximize the production throughput, without sacrificing the customizability of the printed NCS. Further advancements are centered around the room-temperature processed semiconductor material which enables flexible and plastic substrate carriers for the future.

We consider this work as an important step towards the realization of printed neuromorphic computing systems which can support the penetration of pervasive computing systems in future application domains, which require extremely low-cost, green and flexible computing architectures, e.g., for direct processing of sensory data of wearable and smart devices.

## Methods

### Inkjet-printing technology

The transistors were fabricated by inkjet-printable functional inks: ${\hbox {In}_{2}\hbox {O}_3}$ for the semiconductor channel material, composite solid polymer electrolyte (CSPE) for the dielectric substitute and PEDOT:PSS as the electrical conductor for the top-gate contact. For the printing process the Dimatix DMP-2850 inkjet printer was deployed, which supports exchangeable inkjet cartridges. In total three cartridges were used, one for each of the functional materials. For the fabrication of the inorganic n-type electrolyte-gated transistors (EGT), first the semiconductor is printed and annealed at ${400}^{\circ }$ for two hours. In a subsequent step, the electrolyte is printed on top of the semiconductor channel. Finally PEDOT:PSS is printed to provide a top-gate contact. These processing steps are explained in more detail in the Supplementary document. In addition to the top-gate contact, PEDOT:PSS is also printed to obtain resistors for the MAC circuit. The feasible range of printed resistors was found experimentally and is [${100}{\hbox {k}}{\Omega }$,${10}{\hbox {M}\Omega }$].

### Electrical characterization

The electrical characterization was performed at constant humidity level (50%) and room temperature (${295}^{\circ }\hbox {K}$) using a Yokogawa DL6104 digital oscilloscope in combination with a Süss Microtech probe station. For the MAC circuit, transient measurements were performed with a combination of input pulses ranging from ${-1}{\hbox {V}}$ and ${1}{\hbox {V}}$. For the pulse generation two Keithley 3390 waveform generators were utilized. For the measurements of the *inv* and *ptanh* circuit, DC measurements were performed, using the Keithley 3390 waveform generators with a very slow voltage ramping signal, starting at ${-1}{\hbox {V}}$ and ending at ${1}{\hbox {V}}$. The power supply for VDD and VSS for all three measurements were provided by Agilent 4156C Semiconductor Parameter Analyzer.

## Supplementary information

Supplementary information 1.

## Data Availability

Data related to this study are available upon request from the corresponding authors.
