# Supplementary material for: Realization and training of an inverter-based printed neuromorphic computing system
Source: Sci Rep. 2021 May 5;11:9554. doi: 10.1038/s41598-021-88396-0 (PMC8099883; doi:10.1038/s41598-021-88396-0)
Supplement: Supplementary file 1 — Supplementary information 1. [file 41598_2021_88396_MOESM1_ESM.pdf]

Supplementary Information

Realization and training of an inverter-based printed neuromorphic computing system

Dennis D. Weller[1,2], Michael Hefenbrock[3], Michael Beigl[3], Jasmin Aghassi-Hagmann[2,4], and Mehdi B. Tahoori[1,*]

[1] *Chair of Dependable Nano Computing, Karlsruhe Institute of Technology, 76131 Karlsruhe, Germany*
[2] *Institute of Nanotechnology, Karlsruhe Institute of Technology, 76344 Eggenstein-Leopoldshafen, Germany*
[3] *Chair of Pervasive Computing Systems, Karlsruhe Institute of Technology, 76131 Karlsruhe, Germany*
[4] *Institute for Applied Research, Offenburg University of Applied Sciences, 77652 Offenburg, Germany*
[*] *Correspondence to mehdi.tahoori@kit.edu*

1 Inkjet-printing technology

1.1 Fabrication details of the hardware prototypes

For the hardware prototypes a 20mm×20mm ITO-sputtered glass substrate (PGO CEC020S) with a sheet resistance of 20Ω/□ and a layer thickness of 100nm was structured using laser ablation with a Trumpf TruMicro5000 picosecond laser with a infra-red laser wavelength of 1030nm and 2.5W laser power to obtain the passive conductive tracks. After laser ablation, the substrate was cleaned for 20 minutes in an ultrasonic bath from Sonorex Digital 10P containing a 50% Aceton and 50% Isopropanon solution. Next, before semiconductor printing, surface treatment was performed with an oxygen plasmer cleaner from Diener Electronic for 2min. For the EGT fabrication, first the semiconductor ink was printed between the source and drain electrodes of the ITO conductive tracks. After annealing the substrate at 400°C for 2 hours in a Nabertherm P330 muffle furnace, and a two hours ramp from room temperature to 400°C, the sheet resistance of the ITO conductive tracks was about 80Ω/□ and the semiconductor In_2O_3 was obtained. After a cool-down phase, the CSPE was printed covering the drain, source and gate ITO electrodes. Subsequently, PEDOT:PSS was printed to obtain a top-gate contact between the electrolyte and the ITO gate electrode. The EGT printing steps are illustrated in Figure 1. In addition to EGT printing, for the MAC circuit PEDOT:PSS was printed to realize the printed NN weights.

The Microscope photos were taken by a LEICA DMLM microscope. The multiple microscope photos were stitched by GNU Image Manipulation Program (GIMP).

1.2 Ink preparation

For the semiconductor ink, $In_2(NO_3)_3$ (Indium (III) nitrate hydrate, Sigma-Aldrich, 99.9% trace metal basis, MW = 300.83 $\mathrm{gmol^{-1}}$) was dissolved in double-deionized water and glycerol (Merck KGaA, MW = 92.09 $\mathrm{g\,mol^{-1}}$) with a ratio of 4:1 [1]. After stirring for 1 hour, the solution was filtered with 0.2μm polyvinylidene fluoride (PVDF) syringe filter before injecting it in the printer cartridge ink reservoir.

For the CSPE ink, 0.3g PVA (98% poly(vinyl alcohol) hydrolyzed) was dissolved in 6g DMSO (Sigma-Aldrich, Dimethyl sulfoxide anhydrous, MW = 78.13 $\mathrm{g\,mol^{-1}}$, 99.9%), and stirred for 2 hours at 90°C [1]. In parallel, 0.07g of $LiClO_4$, lithium perchlorate (Sigma Aldrich, MW = 106.39 $\mathrm{g\,mol^{-1}}$, 99.99% trace metal basis) was dissolved in 0.63g PC (Propylene carbonate anhydrous, Sigma-Aldrich, 99.7%, 102.09 $\mathrm{g\,mol^{-1}}$) and stirred for 1 hour at room temperature. Both solutions were mixed and stirred until a clear solution was obtained. The solution was then filtered with a 0.2μm PTFE (polytetrafluoroethylene) syringe filter before injecting it into the printer cartridge ink reservoir.

The PEDOT:PSS conductive ink was prepared by mixing 70% PEDOT:PSS (Sigma-Aldrich, Poly(3.4-ethylenedioxythiophene) polystyrene sulfonate, 3.0- 4.0% in H_2O) with 30% ethylene glycol (Sigma-Aldrich, ethylene glycol anhydrous, 99%, MW = 62.07 $\mathrm{g\,mol^{-1}}$) [1]. It was stirred at room temperature until a clear solution was obtained. The solution was then filtered by 0.2μm polyvinylidene fluoride (PVDF) syringe filter before injecting it in the printer cartridge ink reservoir.

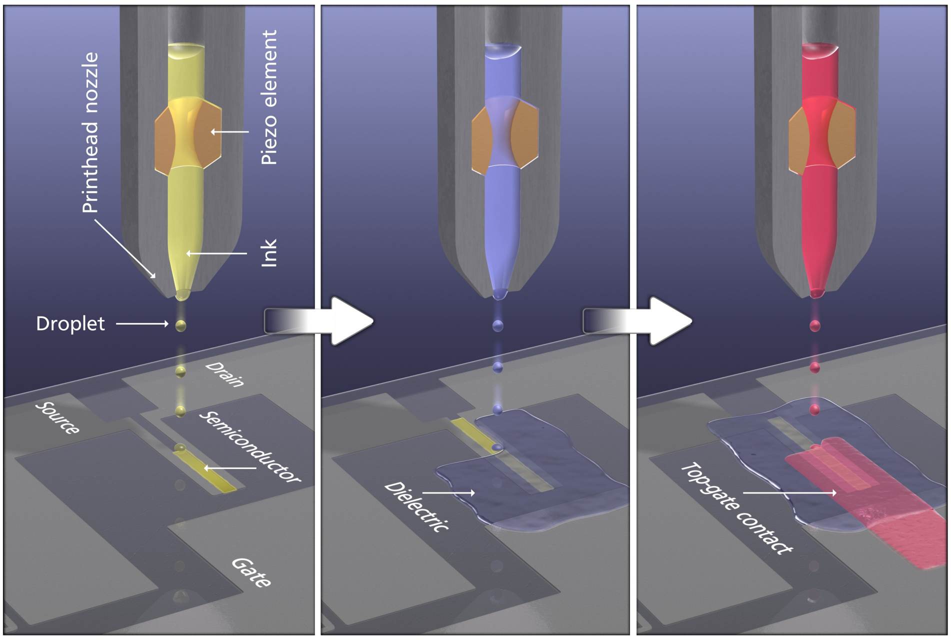

Figure 1: *Inkjet-Printing Process of n-type electrolyte-gated transistors.*

2 Resistors to NN weights calculation

The output voltage V_x of the crossbar architecture depends on the input voltages applied to the resistors (V_i or $\bar{V}_i$ for inverted inputs) and the crossbar resistors R_i, R_b and R_d. These crossbar resistors form a Y-circuit (with V_x in the center) with only linear devices (resistors), thus the output signal V_x can be solved analytically.

The currents through all resistors are computed by Ohm's law: $I_i = (V_i - V_x)/R_i = (V_i - V_x)\ g_i$, or $I_b = (V_{bias} - V_x)/R_b = (V_{bias} - V_x)\ g_b$ for the bias resistor R_b and $I_d = (0V - V_x)/R_d = -V_x\ g_d$, for the decoupling resistor, respectively.

These currents are now summed up according to Kirchhoff's rule:

$$\sum\nolimits_{i=1}^{P} I_i + I_b + I_d = 0 \tag{1}$$

By substituting the calculated currents into Eq. (1), and by solving for V_x, the following equation is obtained:

$$V_x = \frac{\left(\sum_i \frac{V_i}{R_i}\right) + \frac{V_{bias}}{R_b}}{\left(\sum_i \frac{1}{R_i}\right) + \frac{1}{R_b} + \frac{1}{R_d}} \tag{2}$$

To obtain the relation between the hardware- and model level of the NN weights, Eq. (2) can be rewritten in the following form:

$$\begin{aligned} V_x &= \frac{\left(\sum_i \frac{V_i}{R_i}\right) + \frac{V_{bias}}{R_b}}{\left(\sum_i \frac{1}{R_i}\right) + \frac{1}{R_b} + \frac{1}{R_d}} \\ &= \sum_i V_i\ w_i + V_{bias}\ w_b \\ &= \sum_i x_i\ w_i + b \end{aligned} \tag{3}$$

With the synaptic weights abbreviated by:

$$\begin{aligned} w_i &= \frac{\frac{1}{R_i}}{\left(\sum_j \frac{1}{R_j}\right) + \frac{1}{R_b} + \frac{1}{R_d}} \\ &= \frac{g_i}{\left(\sum_j g_j\right) + g_b + g_d} \end{aligned} \tag{4}$$

and the bias weight:

$$\begin{aligned} w_b &= \frac{\frac{1}{R_b}}{\left(\sum_j \frac{1}{R_i}\right) + \frac{1}{R_b} + \frac{1}{R_d}} \\ &= \frac{g_b}{\left(\sum_j g_j\right) + g_b + g_d} \end{aligned} \tag{5}$$

and the decoupling weight:

$$w_d = \frac{\frac{1}{R_d}}{\left(\sum_j \frac{1}{R_i}\right) + \frac{1}{R_b} + \frac{1}{R_d}} = \frac{g_d}{\left(\sum_j g_j\right) + g_b + g_d} \quad (6)$$

Thus, the crossbar output V_x implements the MAC operation of artificial neural networks [2]:

$$a = V_x = \sum_i w_i \ V_i + w_b \ V_{bias} = \sum_i w_i x_i + b$$

As can be obtained from Eqs. (4), (5) and (6), the NN weights are determined by the resistances of the printed crossbar resistors R_i. To achieve one-time programmability of the NN, it is sufficient to vary the resistances R_i by changing the lateral geometries of the resistors according to the pre-trained NN weights obtained from the NN learning procedure. To this end, mapping between NN weights and crossbar resistors can be performed by using Eqs. (4), (5) and (6).

3 Power-aware circuit redesign

In Table 2 of the manuscript, we report on the performance, power consumption and area of the hardware prototypes. Regarding the 'inv'-block (negative ANN weights circuit) there is the possibility to reduce the power consumption by resizing the resistor components without changing the circuit structure itself. To this end, the voltage divider resistances can be scaled up to obtain a low-power design point. To validate this claim, we performed a circuit analysis on an up-scaled 'inv' block simulation-based. By using a scaling factor of 50 for R1/R2 and a factor 22.4/26.6 for R3/R4, the previous resistance values for R1, R2, R3 and R4 (160Ω, 80Ω, 25kΩ, 15kΩ) were up-scaled (8kΩ, 4kΩ, 560kΩ, 400kΩ). As expected, the power consumption of the 'inv' block was reduced from 30W to 770μW, with an increase in delay of 8ms (before 4ms). As the higher resistance values lead to a larger hardware footprint, also the area usage was increased to 56.21mm^2 (before 20.3mm^2). The authors want to note here, that an increase of resistances is also achievable by using a low-conductive-ink-based printed resistor to reduce the resistor area.
As the change of resistances in this low-power 'inv' block impacts also the transfer function, simulations were performed to observe this deviation, as illustrated in Figure 2. From Figure 2 we can observe however, that the behaviour of the circuit is only slightly changed compared to the previously reported high-power 'inv' block, and can still be deployed to realize the negative weights operation.

4 Training printed neural networks

The main challenge for training the printed neural networks arises from the need to take the circuit level constraints, i.e.

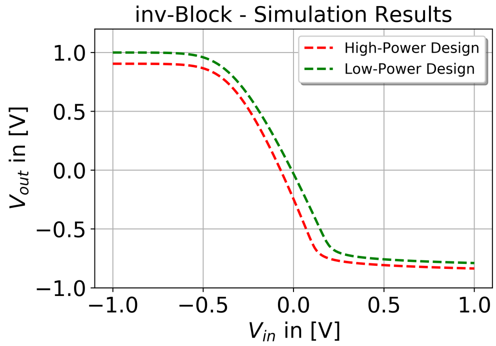

Figure 2: *Simulated transfer curve of the high-power (low resistances) and low-power (high resistances) inv-block.*

feasible conductance ranges of $g_i \in [g_{min}, g_{max}] \cup \{0\}$, the specific realization of negative weights and the activation function into account. To this end, we developed a training framework capable of addressing these characteristics.

To reach an agreement between a model that reflects the physical constraints and still qualifying for gradient-based optimization such as backpropagation [3], we introduce surrogate conductances θ, representing our trainable parameters.

The surrogate conductances encode the value of a respective conductance through their absolute value, i.e. $g_i = \mid \theta_i \mid$, while the sign of θ_i encodes if the input to the respective resistor should be inverted (negative weight).

Using the surrogate conductances, we express the weights through a modified version of Eq. (4) as

$$w_i = \frac{\mid \theta_i \mid}{\sum_j \mid \theta_j \mid + \mid \theta_b \mid + \mid \theta_d \mid}. \quad (7)$$

Furthermore, the dot product $\mathbf{w}^T\mathbf{x} = \sum_i w_i \cdot x_i$ of inputs and weights is expressed through

$$\sum_i w_i \left(x_i \cdot \mathbb{1}_{\{\theta_i \geq 0\}} + \text{inv}(x_i) \cdot \mathbb{1}_{\{\theta_i < 0\}} \right),$$

where inv(x) denotes the inverted input x (see Figure 3 (c) in the main document for the graph of inv(x)) and $\mathbb{1}_{\{\cdot\}}$ denotes an indicator function returning 1 if the respective condition is true, else 0.

After applying the ptanh activation function to the result of the weighted sum operation, we refer to the output as the activation of the neuron.

4.1 The loss function

In neural network training, the loss function guides the learning process and expresses the favorability of the current result. Typical choices for the loss function are, e.g., mean squared error (MSE) or cross-entropy (CE) [4]. In all cases, for some data $\mathcal{D} = \{(\mathbf{x}_n, y_n)\}_{n=0}^{N}$, the loss function measures a distance of the output of the network $f_{\boldsymbol{\theta}}(\mathbf{x}_n)$ for a training instance $\mathbf{x}_n$ to the true value y_n. The parameter $\boldsymbol{\theta}$ thereby denotes all trainable parameters of the network (usually the weights and biases). In our case, $\boldsymbol{\theta}$ refers to the surrogate conductances θ_i, θ_b and θ_d from which we derive the respective conductance values and connections to print.

In a classification setting, the class labels are usually mapped to natural numbers, i.e., $y_n \in \mathbb{N}$ and can then be associated with an index of the neural network output (vector), e.g., $f_{\boldsymbol{\theta}}(\mathbf{x})_j$ denotes the j-th output.

For appropriately training printed neural networks, we need to take their specific characteristics into account. Firstly, due to the bounded weights and activation function, the achievable output range of the printed neural network is limited. Additionally, the range of measurable (and therefore distinguishable) output values needs to be considered.

To respect these limitations, we construct a loss function inspired by the multi-class-hinge-loss [5],

$$L(\boldsymbol{\theta}) = \frac{1}{\mid \mathcal{D} \mid} \sum_{(\mathbf{x},y)\in\mathcal{D}} l(\mathbf{x}, y, \boldsymbol{\theta}),$$

with

$$l(\mathbf{x}, y, \boldsymbol{\theta}) = (m + T - f_{\boldsymbol{\theta}}(\mathbf{x})_y)^+ + (m + \max_{j\neq y} f_{\boldsymbol{\theta}}(\mathbf{x})_j)^+,$$

where $T \in \mathbb{R}^+$ is an implementation-specific measuring threshold, $m \in \mathbb{R}^+$ is a user-defined parameter called margin and $(\cdot)^+ = \max\{0, \cdot\}$.

Using this loss function, a positive loss is incurred if the output activation of the neuron associated with the correct class $f_{\boldsymbol{\theta}}(\mathbf{x})_y$ does not surpass $m + T$ (margin and threshold), or any wrong network output $f_{\boldsymbol{\theta}}(\mathbf{x})_{j\neq y}$ is bigger than $-m$.

For our application, we want to set T to a value above which a signal is measurable and can be distinguished from 0V, while the margin m is a tuning parameter.

4.2 Parameter initialization for learning

It is well known that proper initialization of the parameters can be crucial for the success of the training of neural networks [6]. One reason for this is that unsuitable initializations may lead to unfavorable propagation dynamics which hinder the learning process. For example, if the initialization leads to outputs in the saturation regions of the activation function, gradients close to zero (vanishing gradients) can be observed in the backward pass of backpropagation.

To mitigate these effects, multiple strategies have been developed to find good heuristics for the parameter initialization. In these schemes, the weights are usually drawn uniformly or normally distributed around zero [4], where the scale is related to the number of inputs and/or output connections [6]. Additionally, the scheme may vary based on the activation function [7].

In our case, the scale of the resulting network weights w_i is already directly related to the number of inputs through the coupling constraint of the crossbar (see Eq. (4)). We can therefore simply initialize the surrogate conductances θ_i uniformly around zero with a constant deviation. Since we generally expect few neurons in each layer, the mean of the surrogate conductances may not reflect the expected value of the distribution well (i.e., may be non-zero). To address this, we can center them after initialization by subtracting the empirical mean.

Since the surrogate conductance θ_d (representing g_d) is mainly used for decoupling the weights, it should be set to allow for maximal decoupling which relates to $\theta_d = g_{max}$.

Finally, the initialization of the surrogate conductance for the bias θ_b plays an important role relating to the ptanh activation function. When the input of the activation function is close to zero, which can be expected when the initial weights are small, the ptanh activation function is already rather far in the (negative) saturation region. This will likely yield very similar (negative) outputs for most of the training instances in the beginning of the learning process and makes it harder for the network to separate them. Additionally, the gradients obtained in the saturation region will be small which can hinder the progress of learning.

We try to mitigate this effect by setting the initial bias (see Eq. (5)) to the root of ptanh through:

$$\theta_b = \frac{\text{ptanh}^{-1}(0)}{1 - \text{ptanh}^{-1}(0)} \left(\sum_i \mid \theta_i \mid + \mid \theta_d \mid \right). \tag{8}$$

Through this, the initial inputs to the activation functions across the layers should lead to outputs being closely distributed around zero where better gradients can be obtained and the outputs for different training instances are more diverse.

4.3 Backpropagation for printed neural networks

To train the printed neural network, the classical backpropagation algorithm [3] can be used with a small modification.

In the following, we summarize all parameters (i.e., the surrogate conductances θ_i) of the printed neural network in the vector $\boldsymbol{\theta}$. Then, a gradient descent update at iteration t is realized by

$$\boldsymbol{\theta}_t \leftarrow \text{Proj}\left[\boldsymbol{\theta}_{t-1} - \alpha \cdot \nabla_{\boldsymbol{\theta}} \text{Obj}(\boldsymbol{\theta}_{t-1})\right],$$

where $\alpha \in \mathbb{R}^+$ denotes the learning rate and $\text{Obj}(\boldsymbol{\theta}_{t-1})$ is the training objective for the parameter values at iteration $t-1$. The projection operation

$$\text{Proj}[\boldsymbol{\theta}] = \begin{cases} -g_{max} & \theta < -g_{max} \\ \theta & \theta \in [-g_{max}, g_{max}] \\ g_{max} & \theta > g_{max}, \end{cases}$$

relates to an element-wise projection of the entries θ of $\boldsymbol{\theta}$.

Such simple projections are also commonly referred to as *clipping*.

Note that the training objective can either consist of only the loss function or additionally include penalty terms as in Eq. (9). Additionally, the update step is not limited to only gradient descent, but any gradient-based method can be used to obtain the next iterate, e.g., Adam [8].

Through the projection, all surrogate conductances will lay in the range $[-g_{max}, g_{max}]$. However, only conductances in the range of $[g_{min}, g_{max}] \cup \{0\}$ can be fabricated.

4.4 Projecting infeasible conductances after training

To guarantee feasible conductance values,

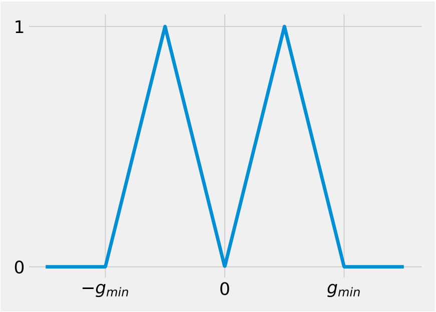

Figure 3: *The penalty function. Using this penalty function, infeasible parameter ranges result in higher values of the overall loss.*

all infeasible conductances in the range of $g_i \in (0, g_{min})$ are projected (rounded) to 0 after training. This relates to not printing the respective resistor. However, this might lead to a degradation in accuracy.

To mitigate this effect, we introduce a penalty function for the infeasible region of $g \in (0, g_{min})$, to encourage the training algorithm to avoid these values.

The penalty function is thereby loosely inspired by penalty methods for weight quantization (see [9]). It is constructed out of two hat functions which are placed in the middle of the infeasible domain with an appropriate width to cover the region $[0, g_{min}]$, i.e.,

$$P(\theta) = \left(1 - \frac{|2\theta - g_{min}|}{g_{min}}\right)^{+} + \left(1 - \frac{|2\theta + g_{min}|}{g_{min}}\right)^{+}.$$

An illustration of the penalty function $P(\theta)$ for a single conductance can be seen in Figure 3.

Through the loss $L(\boldsymbol{\theta})$ and the penalty function $P(\theta)$, a combined training objective can be formulated as

$$\mathrm{Obj}(\boldsymbol{\theta}) = L(\boldsymbol{\theta}) + \frac{\lambda}{\dim(\boldsymbol{\theta})} \sum_{\forall \theta \in \boldsymbol{\theta}} P(\theta), \tag{9}$$

where $\lambda \in \mathbb{R}^{+}$ denotes a tuning parameter and the number of entries $\dim(\boldsymbol{\theta})$ of $\boldsymbol{\theta}$ is used to normalize the influence of different number of parameters for different models. By optimizing the combined objective, the training process is made aware of the impracticability of parameter values in the infeasible range.

4.5 Variation models

There are several sources of variation which can impact the circuit behavior of the proposed analog ANN building blocks. E.g., the MAC circuit is susceptible to resistor variations, while the 'inv' and 'ptanh' circuits are also influenced by fluctuations of the voltage supply, temperature and transistor process variations. As part of our PVT (process, voltage, temperature) variation analysis, we derived variation models for the ANN building blocks, namely the conductances forming the weights, as well as the 'inv' and 'ptanh' circuit, which are described in the following.

4.5.1 Conductance variations

Variations of the conductance state of a resistor can be caused by deviations of the geometrical parameters such as length or area, or by variations of the material-related quantity expressed as the conductivity. If we assume an industrial printing process with perfect control over the alignment of printed resistors, geometrical variations can be neglected and only deviations of the conductivity of the material are present due to differences of the material properties or by environmental fluctuations of temperature and humidity. Based on this assumption, we formulate a conductance variation model with a variation level ϵ, of the following form,

$$g = \bar{g} \cdot (1 + r) \quad \text{with} \quad p(r) = \mathcal{U}(-\epsilon, \epsilon), \tag{10}$$

where $\bar{g}$ denotes the intended conductance and r is a random variable drawn from a uniform distribution with bounds ϵ. Since the conductance variation is assumed independent, the joint variation model for the conductances is simply the product of the individual distributions.

4.5.2 Variation models for the 'inv' and 'ptanh' functions

In order to derive a variation model for the PVT analysis of our pNN, a probabilistic model of the inverters ('inv') and activation function ('ptanh') block are required. Both circuits implement an analog transfer function whose shape is impacted by PVT variations. Depending on the PVT variation corner, variations were injected during circuit simulations of the 'inv' and 'ptanh' blocks. The variation model of the resistances was chosen as described above. For the process and temperature variations of the printed transistors, we used the variation model of [10]. Voltage fluctuations were assumed to be standard normal distributed. Based on these variation models, Monte Carlo circuit simulations were performed and the transfer functions under variation were obtained. Subsequently, for each transfer function and ANN building block ('inv' and 'ptanh'), a parameterized hyperbolic tangent (tanh) model was fitted to the circuit simulation waveforms, as described in the main manuscript (see Eq. (8) and (9)), and the parameters $\boldsymbol{\eta}$ were calculated.

This was repeated 1000 times for each variation level ϵ to obtain a dataset of extracted curve parameters $\boldsymbol{\eta}$ for the inv and the ptanh function under a given variation level ϵ.

The datasets for each ϵ are then modelled through a Gaussian mixture model (see [2]) where the number of components is selected through cross-validation based on the maximum average log-likelihood. The details of the procedure are similar to [10]. The probability distributions of the parameters $\boldsymbol{\eta}$ for a given variation level ϵ obtained this way are referred to as $p(\boldsymbol{\eta})$ in the following.

4.6 Variation-aware training

To mitigate the influence of these variations on the performance of the printed neural networks, we explicitly consider them in the training procedure through the derived variation models. In the classic (nominal) neural

network training the loss function $L(\boldsymbol{\theta})$ on the data is minimized with respect to the parameters $\boldsymbol{\theta}$. However, given conductance variations, the surrogate conductances $\boldsymbol{\theta}$ should be considered as random variables with a distribution reflecting this conductance variation. Additionally, each activation and inverter function may behave differently depending on their sampled parameters $\boldsymbol{\eta} \sim p(\boldsymbol{\eta})$ and can influence the inference and the loss. Consequently, the loss $L(\boldsymbol{\theta}, \boldsymbol{\eta})$ also becomes a random variable. In this case, the new training objective should be to minimize the expected loss under the parameter variations given by our variation models for $\boldsymbol{\theta}$ and $\boldsymbol{\eta}$ through adjusting trainable parameters.

The trainable parameters, which we denote $\boldsymbol{\omega}$ in the following, are thereby the parameters of the joint parameter distribution $p(\boldsymbol{\theta}, \boldsymbol{\eta}; \boldsymbol{\omega})$. This distribution is given by our variation models (for a set variation level ϵ) and influence which parameters are sampled. As an example, consider the entry ω_i of $\boldsymbol{\omega}$ to be the intended nominal surrogate conductance value of θ_i in $\boldsymbol{\theta}$. Depending on which nominal values are aimed for, different samples of $\boldsymbol{\theta}$ are observed. The training objective therefore becomes

$$\min_{\boldsymbol{\omega}} \mathbb{E}_{p(\boldsymbol{\theta}, \boldsymbol{\eta}; \boldsymbol{\omega})}[L(\boldsymbol{\theta}, \boldsymbol{\eta})] = \int L(\boldsymbol{\theta}, \boldsymbol{\eta}) p(\boldsymbol{\theta}, \boldsymbol{\eta}; \boldsymbol{\omega}) d\boldsymbol{\theta} d\boldsymbol{\eta}. \quad (11)$$

Since our variation model for the inv and ptanh function parameters $p(\boldsymbol{\eta})$ (see Section 4.5.2) does not contain any adjustable parameters beside its variation level, we only consider the distribution of $\boldsymbol{\theta}$ as trainable and $p(\boldsymbol{\theta}, \boldsymbol{\eta}; \boldsymbol{\omega}) = p(\boldsymbol{\theta}; \boldsymbol{\omega}) p(\boldsymbol{\eta})$. In other words, the distribution of the surrogate conductances $\boldsymbol{\theta}$ can be influenced by the training algorithm through the choice of $\boldsymbol{\omega}$ (intended surrogate conductance values), while the variation of the inv and ptanh function are independent and cannot be influenced by the network training in our case.

Nevertheless, since the objective function in Eq. (11) (and also its gradients) will usually not have a closed form solution, approximations via, e.g., Monte Carlo estimates through samples from $p(\boldsymbol{\theta}; \boldsymbol{\omega})$ and $p(\boldsymbol{\eta})$ have to be used. While estimating the objective in Eq. (11) (for a given $\boldsymbol{\omega}$) through Monte Carlo samples is straightforward, obtaining an estimate of its gradient with respect to $\boldsymbol{\omega}$ for training, i.e.,

$$\nabla_{\boldsymbol{\omega}} \int L(\boldsymbol{\theta}, \boldsymbol{\eta}) p(\boldsymbol{\theta}; \boldsymbol{\omega}) p(\boldsymbol{\eta}) d\boldsymbol{\theta} d\boldsymbol{\eta},$$

requires techniques for Monte Carlo gradient estimation.

One of these techniques is the pathwise gradient estimator (see [11]). The idea of this estimator is to generate samples from a simple base distribution $p(\mathbf{r})$ and transform them to samples from the desired distribution, here $p(\boldsymbol{\theta}; \boldsymbol{\omega})$, through a differentiable deterministic function $h(\mathbf{r}, \boldsymbol{\omega})$ which is referred to as path. For $p(\boldsymbol{\theta}; \boldsymbol{\omega})$, we can simply use the path $\boldsymbol{\theta} = h(\mathbf{r}, \boldsymbol{\omega}) = (1 + \mathbf{r}) \operatorname{diag}(\boldsymbol{\omega})$ and draw samples from $p(\mathbf{r})$ analogous to Eq. (10) based on the variation level ϵ. The absolute value of the surrogate conductances $|\theta_i|$ will then follows the same distribution as the conductances g, while θ_i inherits its sign from ω_i.

Since $p(\boldsymbol{\eta})$ does not have trainable parameters (i.e., no dependence on $\boldsymbol{\omega}$), it requires no explicit sampling path.

With these reformulations, the gradients of the expected loss can be estimated through

$$\begin{aligned}
\nabla_{\boldsymbol{\omega}} \mathbb{E}_{p(\boldsymbol{\theta}, \boldsymbol{\eta}; \boldsymbol{\omega})}[L(\boldsymbol{\theta}, \boldsymbol{\eta})] &= \nabla_{\boldsymbol{\omega}} \int L(\boldsymbol{\theta}, \boldsymbol{\eta}) p(\boldsymbol{\theta}; \boldsymbol{\omega}) p(\boldsymbol{\eta}) d\boldsymbol{\theta} d\boldsymbol{\eta} \\
&= \int \nabla_{\boldsymbol{\omega}} L(h(\mathbf{r}, \boldsymbol{\omega}), \boldsymbol{\eta}) p(\mathbf{r}) p(\boldsymbol{\eta}) d\mathbf{r} d\boldsymbol{\eta} \\
&= \mathbb{E}_{p(\mathbf{r}) p(\boldsymbol{\eta})}[\nabla_{\boldsymbol{\omega}} L(h(\mathbf{r}, \boldsymbol{\omega}), \boldsymbol{\eta})] \\
&\approx \frac{1}{N} \sum_{n=1}^{N} \nabla_{\boldsymbol{\omega}} L(h(\mathbf{r}^{(n)}, \boldsymbol{\omega}), \boldsymbol{\eta}^{(n)}) \\
&\text{with} \quad \mathbf{r}^{(n)} \sim p(\mathbf{r}), \quad \boldsymbol{\eta}^{(n)} \sim p(\boldsymbol{\eta}),
\end{aligned}$$

where N is the number of samples drawn for the estimation of the gradient. The estimated gradients can then be used in the training described in Section 4.3.

5 Simulation results on benchmark datasets

The following describes the generation of the evaluation results in Table 3 of the main document. We selected nine datasets (eleven classification tasks) from the UCI ML repository [12], namely *Acute inflammation* [13], *Breast Cancer Wisconsin*, *Energy efficiency* [14] (two tasks), *Iris* [15, 16], *Balance Scale*, *Seeds* and *Vertebral Column* (two tasks).

To avoid preprocessing, we use the already prepared versions of the data provided in [17]. The features were additionally normalized to a range of $[0, 1]$ and a train-test-splits with 66% of the data for training and 33% for testing was performed.

Since a 100 % accuracy result is not achievable on all tasks, we try to put the printed neural network results into perspective by also training a (hardware agnostic) reference neural networks for comparable configurations. The reference network uses the same architecture, has no parameter restrictions, can apply weight decay, and uses the standard tanh activation function. Ideally, we expect the printed neural network to achieve a similar performance to the reference network on all tasks.

5.1 Evaluation metric

Generally, classification tasks are most naturally evaluated using the accuracy metric (the fraction of correctly classified instances). However, due to measuring limitations, we need to take aspects of signal separation into account. We therefore evaluate our circuit with a stricter version of the accuracy called the "Measuring-aware accuracy" (MaA). Assuming the class variable y is encoded as natural numbers $y \in \mathbb{N}$, the MaA is defined as

$$\sum_{(\mathbf{x}, y) \in \mathcal{D}} \frac{\mathbb{1}_{\{i == y\}} \cdot \mathbb{1}_{\{f_{\boldsymbol{\theta}}(\mathbf{x})_y > T\}} \cdot \mathbb{1}_{\{\forall j \neq i \mid f_{\boldsymbol{\theta}}(\mathbf{x})_j < 0\}}}{|\mathcal{D}|}, \quad (12)$$

with $i = \operatorname{argmax}_j f_{\boldsymbol{\theta}}(\mathbf{x})_j$ and $\mathcal{D}$ denoting the evaluation data. Compared to the standard accuracy, which would only consider the first condition $\mathbb{1}_{\{i == y\}}$, the measuring-aware accuracy also requires the output of the neural network to exceed the measurably threshold T with

$\mathbb{1}_{\{f_{\boldsymbol{\theta}}(\mathbf{x})_i > T\}}$ and requires all other outputs to be below 0V through $\mathbb{1}_{\{\forall j \neq i \mid f_{\boldsymbol{\theta}}(\mathbf{x})_j < 0\}}$. The proposed evaluation metric is therefore stricter than the standard accuracy and additionally takes the output signal strength and separation into account.

5.2 Hyperparameter configuration and learning

All neural networks in the following are implemented using *pytorch* [18]. For all experiments, we choose the same architecture and training setup of $\#inputs - 4 - 3 - \#classes$ neurons with activation functions after each layer (including the output) and trained them for 200 (full-batch) steps with halving the learning rate every 50 epochs. Additionally, the training is stopped preemptively if a configuration does not achieve an improvement over the baseline after 50 epochs or does not improve over 20 consecutive updates (early stopping).

For better numerical stability, we normalize the values of the surrogate conductance parameters θ to the range of $[0.01, 1]$ representing $R_{min} = 100\text{k}\Omega$ and $R_{max} = 10\text{M}\Omega$. Note that this does not influence the calculations and we can readily retrieve the respective conductance values. For the initialization, the surrogate conductances θ_i relating to weights w_i, are drawn uniformly as $\theta_i \sim \mathcal{U}[-0.01, 0.01]$, while $\theta_b = g_{max}$ and θ_d is initialized as described in Eq. (8). The parameter θ_d relating to the resistance g_d was only adjusted in variation-aware training, while staying fixed at $\theta_d = g_{min}$ when training without variation. The rational behind this is for all neurons to profit as much as possible from the decoupling effect. However, under variation, other values for θ_d may be more beneficial, so choosing the value for the parameter is left to the training routine in this case.

The measuring threshold was set to $T = 100\text{mV}$ while the margin m is considered a hyperparameter. It is therefore selected through a grid-search routine with the values of $m \in \{0.0, 0.1, \cdots, 0.9, 1\}$ alongside the initial learning rate $\alpha \in \{0.001, 0.1, 1\}$ and the penalty parameter $\lambda \in \{0, 0.001, 0.01, 0.1\}$. Additionally, all configurations are run with 10 different random initializations. Since the reference network does not use the penalty function of the pNN, its coefficient λ is used as a weight decay coefficient here. Furthermore, each layer in the reference network is initialized through $w, b \sim \mathcal{U}[-\sqrt{\#inputs^{-1}}, \sqrt{\#inputs^{-1}}]$ according to the *pytorch* default for linear layers.

We report the test set results of the best pNN and the best reference network for each dataset. The best network is thereby selected over $3 \times 4 \times 11 \times 10 = 1320$ (α, λ, m, seed) configurations for each dataset separately. The selection for the best model is done based on the maximum training MaA (see Eq. (12)). For variation-aware training, the training variation was set equal to ϵ. For each dataset, the best configuration from the (nominal) grid-search was used and retrained 10 times (different seeds) with gradient estimates using $N = 100$ samples. The best network was then selected as the network achieving the maximum average training MaA over 100 sampled parameter sets of $\boldsymbol{\theta}$ and $\boldsymbol{\eta}$. As the optimization algorithm, Adam [8] was used with the parameter configuration recommended by the authors ($\beta_1 = 0.9$, $\beta_2 = 0.999$ and $\epsilon = 10^{-8}$).

Finally, note that in general better results might be achievable through more careful hyperparameter tuning and architecture selection on a per dataset basis.

References

1. Marques, G. *et al.* Influence of humidity on the performance of composite polymer electrolyte-gated field-effect transistors and circuits. *IEEE Transactions on Electron Devices* **66,** 2202–2207 (2019).
2. Bishop, C. *Pattern recognition and machine learning* (Springer, 2006).
3. Rumelhart, D., Hinton, G. & Williams, R. Learning representations by back-propagating errors. *Nature* **323,** 533–536 (1986).
4. Goodfellow, I., Bengio, Y. & Courville, A. *Deep Learning* `http://www.deeplearningbook.org` (MIT Press, 2016).
5. Crammer, K. & Singer, Y. On the algorithmic implementation of multiclass kernel-based vector machines. *Journal of machine learning research* **2,** 265–292 (2001).
6. Glorot, X. & Bengio, Y. *Understanding the difficulty of training deep feedforward neural networks* in *Proceedings of the Thirteenth International Conference on Artificial Intelligence and Statistics* **9** (PMLR, 2010), 249–256. `http://proceedings.mlr.press/v9/glorot10a.html`.
7. He, K., Zhang, X., Ren, S. & Sun, J. *Delving Deep into Rectifiers: Surpassing Human-Level Performance on ImageNet Classification* in *2015 IEEE International Conference on Computer Vision (ICCV)* (2015), 1026–1034.
8. Kingma, D. P. & Ba, J. Adam: A Method for Stochastic Optimization. *arXiv e-prints,* arXiv:1412.6980. arXiv: `1412.6980 [cs.LG]` (Dec. 2014).
9. Wang, Y., Wen, W., Song, L. & Li, H. H. *Classification accuracy improvement for neuromorphic computing systems with one-level precision synapses* in *2017 22nd Asia and South Pacific Design Automation Conference (ASP-DAC)* (2017), 776–781.
10. Rasheed, F., Hefenbrock, M., Beigl, M., Tahoori, M. & Aghassi-Hagmann, J. Variability modeling for printed inorganic electrolyte-gated transistors and circuits. *IEEE Transactions on Electron Devices* **66,** 146–152 (2018).
11. Mohamed, S., Rosca, M., Figurnov, M. & Mnih, A. Monte Carlo Gradient Estimation in Machine Learning. *Journal of Machine Learning Research* **21,** 1–62. `http://jmlr.org/papers/v21/19-346.html` (2020).
12. Dua, D. & Graff, C. *UCI Machine Learning Repository* 2017. `http://archive.ics.uci.edu/ml`.

13. Czerniak, J. & Zarzycki, H. *Application of rough sets in the presumptive diagnosis of urinary system diseases* in *Artificial Intelligence and Security in Computing Systems* (Kluwer Academic Publishers, 2003), 41–51.

14. Tsanas, A. & Xifara, A. Accurate quantitative estimation of energy performance of residential buildings using statistical machine learning tools. *Energy and Buildings* **49,** 560–567 (2012).

15. Anderson, E. The species problem in Iris. *Annals of the Missouri Botanical Garden* **23,** 457–509 (1936).

16. Fisher, R. The use of multiple measurements in taxonomic problems. *Annals of eugenics* **7,** 179–188 (1936).

17. Fernández-Delgado, M., Cernadas, E., Barro, S. & Amorim, D. Do we need hundreds of classifiers to solve real world classification problems? *The Journal of Machine Learning Research* **15,** 3133–3181 (2014).

18. Paszke, A. *et al. Automatic differentiation in PyTorch* in *NIPS-W* (2017).
